# Impact of proximal tibio-fibular joint injury on posterolateral corner knee reconstructions, strategies for achieving knee stability in combined injuries: a systematic review

**DOI:** 10.1007/s00590-025-04483-2

**Published:** 2025-09-02

**Authors:** Javier Faus-Cotino, Ainhoa Amarilla-Irusta, Jose Antonio Guerrero, Aingeru Sarriugarte, Isabel Andia

**Affiliations:** 1 Department of Traumatology and Orthopedic Surgery, Cruces University Hospital, 48903 Barakaldo, Spain; 2 Biocruces Bizkaia Health Research Institute, Cruces University Hospital, 48903 Barakaldo, Spain; 3 Immunopathology Group, Biocruces Bizkaia Health Research Institute, Cruces University Hospital , 48903 Barakaldo, Spain; 4 Department of General Surgery , Cruces University Hospital, 48903 Barakaldo, Spain

**Keywords:** Posterolateral corner, Proximal tibiofibular joint, Multiligament knee injury

## Abstract

**Purpose:**

Combined injuries of the posterolateral corner (PLC) and proximal tibiofibular joint (PTFJ) are uncommon but can lead to significant knee instability if not properly managed. While anatomical reconstruction techniques are well defined for isolated PLC injuries, the optimal strategy for managing associated PTFJ instability remains unclear. This review aims to evaluate surgical approaches and outcomes for combined PLC and PTFJ injuries.

**Methods:**

A systematic review was conducted in accordance with PRISMA guidelines. Studies were included if they described surgical management of PLC and PTFJ injuries. Data were extracted regarding patient characteristics, injury mechanisms, surgical techniques, fixation methods, and clinical outcomes. Due to heterogeneity in study design and outcome reporting, a narrative synthesis was performed.

**Results:**

Nine studies met inclusion criteria: one cadaveric biomechanical study, three retrospective series, and five case reports. Most injuries were due to high-energy trauma and commonly associated with multiligament knee injuries. PTFJ stabilization techniques included cortical screw fixation, K-wires, suture constructs, and ligament reconstruction. Despite variability in technique, outcomes were generally favorable when PTFJ instability was addressed in conjunction with PLC reconstruction. Functional scores were reported in only two studies. Documented complications included peroneal nerve palsy, hardware-related pain, infection, and arthrofibrosis.

**Conclusion:**

PTFJ stability plays a critical role in the success of PLC reconstruction. Stabilizing the PTFJ—regardless of technique—appears essential for restoring knee stability. Given the low methodological quality and heterogeneity of current evidence, further prospective studies with standardized protocols are necessary to guide optimal management of these complex injuries.

## Introduction

The posterolateral corner (PLC) of the knee is a critical joint stabilizing region, it is composed by three primary stabilizers (lateral collateral ligament (LCL), popliteofibular ligament (PFL), and popliteus tendon (PT)), and several secondary stabilizers (arcuate ligament, biceps femoris, lateral head of gastrocnemius, or iliotibial band) [1–3]. This region plays a fundamental role in resisting varus stress, external tibial rotation, and maintaining overall knee stability. According to this, injury usually involves a direct varus stress, or a non-contact hyperextension and external rotation force [1]. Historically referred to as the “dark side of the knee,” the PLC has long posed significant challenges due to its intricate anatomy, limited understanding, and historically poor treatment outcomes. While advances in anatomic reconstruction techniques have led to improved results, achieving a balance between accurately replicating the native anatomy and ensuring safe, effective reconstruction remains a persistent challenge [1, 4].

Incidence of PLC injuries ranges from 16 to 28% among patients with severe ligamentous knee trauma. Interestingly, only 28% of these injuries occur in isolation, with the majority being associated with cruciate ligament injuries as part of complex multi-ligament knee injuries (MLKI) [4, 5].

In the context of MLKI, often resulting from high-energy trauma, a comprehensive evaluation of the knee is essential. This evaluation should encompass not only the cruciate and collateral ligaments, but also the PLC structures and the frequently forgotten proximal tibiofibular joint (PTFJ) [5, 6]. Despite PTFJ critical role in mechanical stability, as proximal fibula serves as the attachment site for PLC key structures (LCL, PFL, biceps femoris tendon, and arcuate ligament) [7], proximal tibiofibular joint has received significantly less attention in the literature compared to the tibiofemoral and patellofemoral joints. Although PTFJ instability is rare, accounting for less than 1% of all knee injuries, it is seen in up to 9% of MLKI cases. Injuries to PTFJ joint generally occur during knee flexion, because of fibular head anterior migration due to relaxation of lateral collateral ligament and biceps femoris tendon, facilitating PTFJ lesion and fibular dislocation in anterolateral direction [3, 8]. Notably, this PTFJ instability frequently occurs alongside PLC injuries, which complicates treatment due to the limited working space around the fibular head, the restricted bone availability, and the risk of graft or implant collision, making concurrent treatment of PLC and PTFJ instability technically demanding [3, 9].

Treatment strategy for isolated posterolateral corner injuries largely depends on severity. Low-grade injuries, characterized by less than 10 mm of gapping or less than 10° of external rotation asymmetry, are often managed non-operatively [4]. In contrast, high-grade injuries require surgical intervention to avoid persistent posterolateral instability and increased stress on a potentially reconstructed cruciate ligament, which may promote cruciate graft failure. Over the years, surgical techniques for PLC injuries have evolved, with a growing preference for anatomical reconstructions [1, 4, 10]. Reconstruction techniques can be categorized into fibular-based methods (including Larson and Arciero procedures), and tibiofibular-based methods (including LaPrade reconstruction technique). Biomechanical studies have shown that both LaPrade and Arciero approaches are effective in restoring varus and rotational stability, but neither is able to restore knee stability in combined PLC and PTFJ injuries [4, 10, 11].

Regarding isolated PTFJ injuries, prompt treatment is critical to avoid chronic instability. In the context of acute dislocation, closed or open reduction may be employed, and temporary internal fixation is generally recommended [3, 12]. In isolated PTFJ instability, treatment options include direct repair, ligament reconstruction, screw fixation, or suspensory devices [7, 8, 13]. However, when PTFJ instability is combined with PLC injuries, many of these techniques face limitations, requiring alternative approaches [9].

This study aims to fill the gap in the literature by systematically reviewing the surgical solutions proposed for combined PLC and PTFJ injuries. We will evaluate the anatomical and biomechanical considerations necessary to ensure knee stability in these complex cases. By examining existing techniques and their outcomes, we aim to provide a comprehensive understanding of how PTFJ stability influences PLC reconstruction success and explore strategies to optimize surgical interventions. Through this review, we aim to offer insights into best practices for addressing this challenging injury combination and contribute to the development of more effective, evidence-based treatment protocols.

## Materials and methods

A systematic literature review was conducted following the PRISMA (Preferred Reporting Items for Systematic Reviews and Meta-Analyses) guidelines where applicable. Given the limited number of high-quality studies and the predominance of case reports and small case series, a narrative synthesis was chosen as the most appropriate methodology. This approach allowed for critical evaluation and contextual interpretation of heterogeneous data, including variations in patient populations, surgical techniques, fixation methods, outcome measures, and follow-up durations.

### Search strategy

A search of PubMed, Web of Science and Cochrane Library was conducted by two different authors (A, and B), covering the available literature from 2005. Search strategy included all published literature, without discrimination attending to study design.

Literature search criteria in databases included the use of the following Boolean search terms:

- Posterolateral corner AND tibiofibular.
- Posterolateral AND tibiofibular.
- PLC AND tibiofibular.

### Eligibility criteria and data extraction

**Fig. 1:**
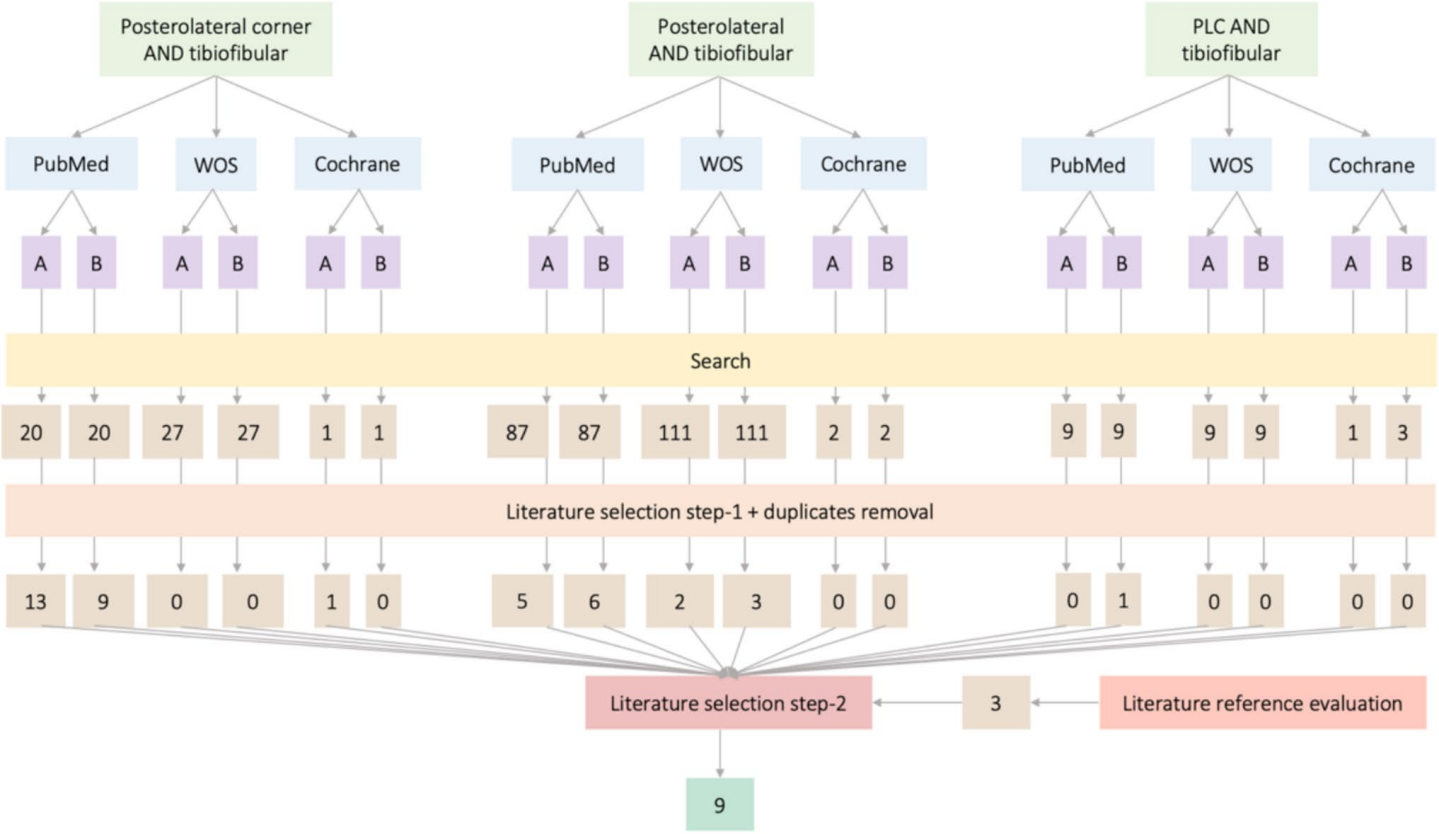
Step-by-step search and selection process flow-diagram

Literature selection was conducted subsequently using a two-step approach (Fig. 1) by applying the following selection criteria:

- *Literature selection step-1* Performed independently by author A and B. Involved the analysis of study title and abstract, inclusion criteria were literature regarding concomitant pathology of posterolateral corner of the knee plus proximal tibiofibular injury. Exclusion criteria included literature material non corresponding to concomitant pathology of posterolateral corner of the knee plus proximal tibiofibular injury. In the event of disagreement between authors, the literature material continued to step-2 of the selection process.
- *Literature selection step-2* Carried out by author A, and performed by full text analysis of the previously selected literature by authors A and B. Inclusion criteria addressed to concomitant pathology of posterolateral corner of the knee and tibiofibular joint requiring surgical treatment. Exclusion criteria included literature material non corresponding to this aim.

Among the selected literature following step-2, individual references were analyzed for detecting any literature material subsidiary to be included.

**Fig. 2:**
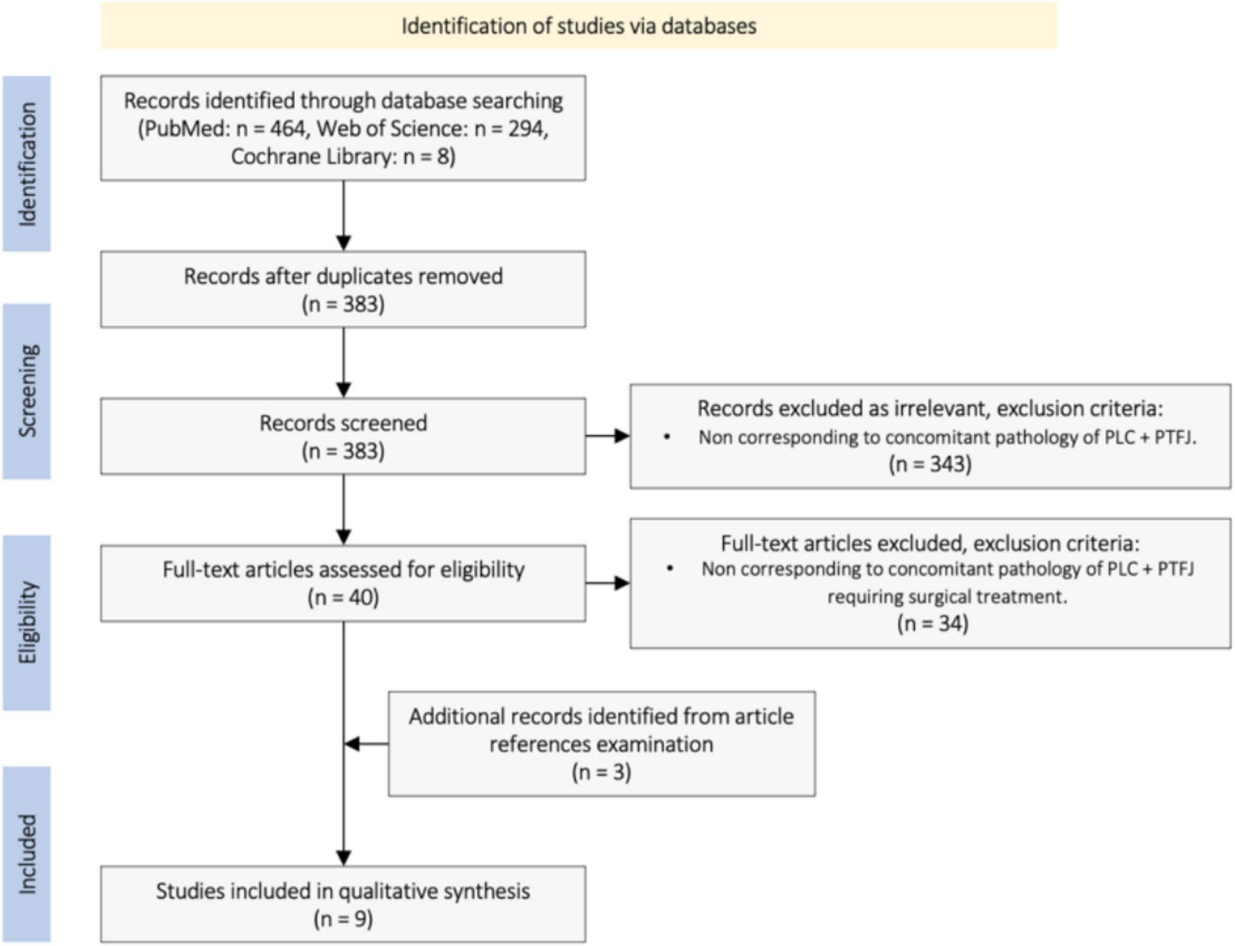
Search and selection process, PRISMA flow-diagram

After application of step-2 selection criteria by author A, plus literature duplicates removal, 9 studies were selected. Attempting to avoid missing of potentially valuable non-identified literature, step-2 selected studies references were individually examined by author A using the same time frame, search and selection criteria, obtaining 3 studies to be selected by using step-1 selection criteria, none of them achieved step-2 selection criteria. A total of 9 studies were selected after full application of search plus selection criteria, individual features of each and results synthesis are described in Results section (Tables 1 and 2). Full search and selection process following PRISMA guidelines is exposed in Fig. 2.

## Results

A total of nine studies met the inclusion criteria, comprising one cadaveric biomechanical investigation, three retrospective clinical series, and five case reports.

Study characteristics, including patient demographics, injury mechanisms, and associated ligamentous injuries, are summarized in Table 1. Most patients sustained high-energy trauma, primarily motor vehicle accidents, with a consistent association between posterolateral corner injuries and proximal tibiofibular joint instability. The diagnosis of PTFJ instability varied across studies, utilizing intraoperative assessment, clinical examination, and imaging modalities such as MRI.

**Table 1:** Study characteristics and pre-surgical data

References	N knees w/PTFJ instability	N knees w/PTFJ instability + PLC injury	N of knees w/PTFJ instabiity + PLC injury treated w/surgery	PTFJ instability definition	Age, sex, side	Injury mechanism	Injured structures
Treme [11]	16	16	16	PTFJ controlled section in cadaveric knee	8 cadavers, mean 78,8 y.o, range 55–95, males. Nr	Controlled section	Sequential controlled section of structures: PLC, PTFJ, ACL
Burke [14]	16	Nr	Nr	Clinical examination + MRI (11 patients)	Mean 31,25 y.o. Nr. Nr	5 motor vehicle accidents. 2 pedestrian vs motor vehicle. 3 non-traumatic. 1 trauma ep (n-e). 1 childhood trauma (n-e)	1 patient: ACL, multiple pelvic + lower extremity fx 2 fx of fibular head Several patients with PLC injury (not specified)
Jabara [7]	12	10	10	Dislocated/dislocatable joint during examination under anesthesia or operative exposure	Mean 32 y.o, range 15–63. 9 males, 3 females. Nr	9 car vehicle accident, 2 motorcycle accident, 1 industrial accident	Among 12 patients: 7 ACL, 3 PCL, 9 LCL, 10 PT, 3 MCL, 5 il femur fx, 4 cl femur fx 1 midfoot fx, 1 cl ankle fx, 5 il proximal tibia fx, 1 il tibia fx, 1 sacrum fx, 1 olecranon fx, 1 il radius fx, 1 cl radius fx, 1 cl acetabulum fx, 2 il patella fx, 1 cl patella fx 1 il below-knee amputation, 1 il pelvis fx,
Sabat [15]	9	9	9	Ballotment test (90° knee flexion and translation PTFJ compared to cl), in outpatient and under anaesthesia	Mean 31 y.o range 24–45. 8 males, 1 female. Nr	9 motor vehicle accident	6 patients: PCL, PTFJ instability 2 patients: ACL, PCL, PTFJ instability 1 patient: ACL, PCL, MCL, PTFJ instability
Sreesobh [13]	2	2	2	Nr	Patient 1: 28 y.o, male, right. Patient 2: 22 y.o, male, nr	Patient 1: motorcycle accident. Patient 2: motorcycle accident	Patient 1: LCL, biceps tendon, PCL, lateral capsule, PTFJ disruption, il open femur fx, il open tibia fx, il PTFJ dislocation, il patellar tendon avulsion Patient 2: inferior dislocation of PTFJ, CPE lesion, right femur fx, right tibia fx, CPE il injury
Porrino [3]	2	2	2	MRI evaluation	Patient 1: 43 year-old, male, right. Patient 2: 19 year-old woman	Patient 1: motorcycle accident. Patient 2: car vehicle accident	Patient 1: knee subluxation, open patella fx, conminuted medial tibial plateau fx, conminuted proximal fibular shaft fx, PTFJ dislocation, ACL complete tear, complete proximal avulsion of PCL, complete distal avulsion MCL, complete tear of proximal LCL, lateral capsular avulsion, medial meniscus tear. Patient 2: open fx left iliac wing, open fx-dislocation left ankle, right radius and ulna fx, degloving left thigh, c2 vertebral fx, disruption of anterior PTF ligamentous capsule, partial tear of ACL, partial avulsion distal PT, rupture of medial and lateral patellar retinaculum
Yuen [9]	1	1	1	PTFJ instability under anesthesia examination	Nr	Nr	PLC, PTFJ
Williams [16]	1	1	1	PTFJ surgical examination	12 y.o, male, left	Sports, non-contact football injury	LCL tear, moderate strain of the PT, proximal posterior tibiofibular tear, ACL avulsion fracture medial tibial eminence
Zeiton [18]	1	1	1	Nr	45 year-old, male, left	Motor vehicle accident	Posterior fibula dislocation (PTFJ and ankle), fx of proximal fibula head, LCL, biceps femoris tendon avulsion (distal insertion)

*ACL* anterior cruciate ligament, *Cl* contralateral, *CPE* common peroneal nerve, *Ep* episode, *Fx* fracture, *Il* ipsilateral, *LCL* lateral collateral ligament, *MCL* medial collateral ligament, *MRI* magnetic resonance imaging, *N-e* not specified, *Nr* non-reported, *PCL* posterior cruciate ligament, *PLC* posterolateral corner, *PT* popliteus tendon, *PTFJ* proximal tibiofibular joint, *W/* with, *Y.o* years old.

Surgical strategies varied considerably among the included studies (Table 2). In the cadaveric study, Treme et al. [11] evaluated the biomechanical performance of the Arciero and LaPrade PLC reconstruction techniques under sequential sectioning conditions. While both techniques restored native joint laxity in isolated PLC-deficient specimens, neither was able to maintain knee stability when PTFJ instability was introduced, highlighting the biomechanical interdependence between the two anatomical regions.

**Table 2:** Surgery-related data

References	Surgery timing from injury	Position, incision, approach (open, arthroscopy, combined)	CPE management	Surgical procedures performed	Grafts required	Tibiofibular stabilizing technique
Treme [11]	Nr, cadaveric study	Nr. Cadaveric study, skin and subcutaneous fat removed. Open	Nr	8 Arciero rec, fixation with polyether ether ketone biotenodesis interference secrews (PEEK). 8 Laprade rec	8 semitendinosus autograft for 8 Arciero rec 8 slipt Achilles autograft with 9 × 20 mm bone plugs for 8 Laprade rec	No PTFJ stabilization technique
Burke [14]	Nr	Nr	Nr	11 fiberwire suture construct (ThightRope, Arthrex). 5 screw fixation. Nr techniques attending to other lesions	Nr	11 fiberwire suture construct (ThightRope, Arthrex). 5 screw fixation
Jabara [7]	Mean 6,3 days range 1–22	Supine. Lateral. Open + arthroscopic evaluation	Identified and dissected for mobilization and protection during surgery	ACL (4 rec,1 repair, 1 non-operative), PCL (7 rec). LCL (3 rec, 6 repair). PT (7 rec, 2 repair. MCL (3 repair). Medial meniscus (4 repair, 1 partial meniscectomy). Lateral meniscus (3 repair, 1 partial meniscectomy). PTFJ joint (10 ORIF, 2 none)	2 patients hamstring autograft. 3 patients soft tissue allograft	Cortical screws: 3,5 mm (4 patients) or 4,5 mm (6 patients),across four cortices. 1 knee lateral-sided bony avulsion from femur contained insertion of LCL, popliteus and lateral capsule, fixation of bony fragment stabilized TF joint
Sabat [15]	Mean time 5 months range 3–8months	Nr. Nr. Open, arthroscopically assisted	Nr	9 knees: PTFJ fixation with 4 mm cancellous cannulated screw. 3 knees: Larson. PTFJ fixation with 2 mm K-wire, 4 mm cannulated screw with washer from fibylar head to proximal tibia crossing 3 cortices directed from the lateral surface of fibular head center in a 45° angle	1: PCL (il st + gr) 2: PCL (il st + gr) 3: PCL (il st + gr), Larson (cl st) 4: ACL (cl st + gr), PCL (il st + gr) 5: PCL (il st + gr) 6: PCL (il pl), Larson (il st) 7: ACL (il pl), PCL (cl pl), MCL (il st)) 8: ACL (il st + gr), PCL (cl pl) 9: PCL (il pl), Larson (il st)	Stabilization with K-wires, fixation with 4mm cancellous cannulated screw with washer
Sreesobh [13]	Nr	Nr	Nr	Patient 1: femoral nailing, tibial external fixator. Trans-knee amputation 7 days from injury. Patient 2: tibial nailing, replacement by external fixator	Nr	Nr
Porrino [3]	Patient 1: nr. Patient 2: 3 weeks after trauma	Nr	Nr	Patient 1: PTFJ reduction and stabilization with 2 mm K-wire prior to definitive fixation with 3,5 mm cortical screw with a washer transfixing PTFJ. Arthroscopic ACL reconstruction with hamstring autograft, reattachment of PCL, repair of MCL superficial and deep components, repair of LCL and lateral capsule, partial medial meniscectomy. Patient 2: reattachment of popliteus tendon to femur with suture anchor, PTFJ fixation with single cortical screw	Patient 1: hamstring autograft for ACL	Patient 1: 3,5 mm cortical screw with washer. Patient 2: cortical screw
Yuen [9]	Nr	Supine. Lateral. Open	Identified and neurolyzed (decompressed on nerve entry into lateral leg compartment)	PLC corner rec, single fibular tunnel and two femoral tunnels (LCL and PT), PTFJ stabilization (Ultrabraid® suture anchors through fibular tunnel and tibial fixation over soft tissue attachment of PTFJ, fixation with 5,5 mm Fooprint Ultra® anchors (Smith and Nephew))	Nr	Nr
Williams [16]	Nr, first medical attention 3 days post-trauma	Nr. Lateral. Open	Neurolysis	Physeal-sparing PLC (near-anatomic reconstruction of LCL and proximal posterior tibiofibular ligament), ACL repair. Femoral anatomic rec tunnel, fibular distal and horizontal tunnel avoiding fibular physis, tibial rec tunnel for proximal posterior tibiofibular ligament. ACL avulsion (fixation with 4 non-absorvable sutures FiberWire, Arthrex + cortical button)	Semitendinous allograft for LCL and posterior TF ligament	Posterior TF ligament reconstruction
Zeiton [18]	Nr	Nr. Lateral. Open	Identified and protected	Ankle sindesmosis fixation (3 tetracortical fully threaded screws through 5-hole third tubular plate), PTFJ fixation (2 mm K-wire + syndesmosis tight rope), LCL rec, repair of biceps femoris tendon (suture anchor TwinFix® Smith–Nephew) to fibula head	Nr	Fixation of PTFJ, nr technique

*ACL* anterior cruciate ligament, *Cl* contralateral, *CPE* common peroneal nerve, *Gr* gracilis, *Il* ipsilateral, *LCL* lateral collateral ligament, *MCL* medial collateral ligament, *Nr* non reported, *PCL* posterior cruciate ligament, *Pl* peroneus longus, *PT* popliteus tendon, *PTFJ* proximal tibiofibular joint, *Rec* reconstruction, *St* semitendinosus, *TF* tibiofibular.

In clinical series, various fixation methods were employed to address PTFJ instability. Burke et al. [14] reported the use of TightRope® suture-button constructs and cortical screws, while Jabara et al. [7] utilized cortical screws across all four cortices to stabilize the joint. In both series, PLC reconstruction techniques were performed concurrently, tailored to the extent and nature of injury. Sabat et al. [15] applied a combination of K-wires and cannulated screws for PTFJ stabilization in a cohort of patients undergoing single-stage multiligament knee reconstruction, including procedures such as Larson reconstruction and transtibial femoral tunnel fixation.

Among the case reports, novel technical adaptations were described. Yuen et al. [9] proposed a combined PLC and PTFJ stabilization technique using Ultrabraid® sutures anchored through a single fibular tunnel, aiming to reduce the risk of tunnel convergence. In a skeletally immature patient, Williams et al. [16] employed a physeal-sparing reconstruction using a single semitendinosus allograft to address both LCL and proximal posterior tibiofibular ligament insufficiencies. These techniques were reported to restore joint stability without growth plate compromise or structural collision.

Postoperative outcomes and complications were inconsistently reported across studies (Table 3). Jabara et al. [7] noted no recurrence of PTFJ instability following surgical fixation and documented meaningful improvements in joint stability and functional scores, with mean Lysholm and IKDC scores of 75 and 58, respectively, at an average follow-up of 32 months. Sabat et al. [15] observed satisfactory clinical outcomes, although several patients experienced persistent grade 1 posterior laxity or terminal flexion limitations. Despite these minor deficits, all patients in their cohort reported satisfaction with surgical results. Functional outcomes in the case reports were typically anecdotal or incompletely documented, though some authors reported return to jogging or sport by six months postoperatively.

**Table 3:** Post-surgery data

References	Post-surgery complications	Postoperative concomitant measures	Surgery outcomes referred	Post-surgery questionnaire	Follow up
Treme [11]	Nr	Nr	Arciero rec and Laprade rec, equally effective restoring stability in varus angulation and external rotation to knee with PLC injury, neither rec restores knee stability when combined PLC + PTFJ injury. No significant difference in external rotation or varus angulation when ACL sectioned	Nr	Nr
Burke [14]	Nr	Nr	Nr		Nr
Jabara [7]	1 failed PLC reconstruction, 1 proximal tibiofibular screw removal secondary to pain over screw head, 1 deep infection, 1 closed manipulation secondary to arthrofibrosis and loss of ROM	Mechanical deep vein thrombosis prevention and chemical prophylaxis. Toe-touch weight-bearing, and bracing. Passive flexion and weightbearing in the brace began at 2–4 weeks post-surgery. Active flexion allowed at 3 months. Hamstring strengthening delayed until 3–4 months. Full return to activities at 9–12 months and when strength was 80–85% of the nonoperative leg determined clinically	No proximal tibiofibular joint inestability recurred at latest follow up. No p complained of ankle stiffness or pain, knee stability in 10 p restored to grade 1 or less in all treated ligaments. 1 p remained with grade 2 PCL instability, and 1 p remained with grade 2 instability of PCL despite revision. ROM obtained at referred follow up (p 1, follow up 36 months, 5–105°. p 2, 33 m, 5–120°. p 3, 61 m, 0–130°. p 4, 48 m, 0–130°. p 5, 36 m, 0–115°. p 6, 24 m, 5–100°. p 7, 37 m, 0–120°. p 8, 24 m, 0–135°. p 9, 30m, 0–130°. p 9, 30 m, 0–130°. p 10, 12 m, 5–125°. p 11, 12 m, 0–130°. p 12, 36 m, 0–130°)	10 patients, mean Lysholm score 75 (range 54–95), mean IKDC score 58 (range 22–78)	Mean 32 m, minimum 12 m, range 12–61 m
Sabat [15]	Nr	Molded knee hinge brace use. Patients with ACL + PCL: non-weight bearing for 3 weeks, then toe-touch weight bearing for 3 weeks, full weight bearing after 6 weeks Patients with PCL + PTFJ: allowed toe-touch weight bearing after 4 weeks with brace. At 3 weeks straight leg raising with no weights. At 6 weeks initiation of static quadriceps exercises, ankle bumps, prone flexion, and closed chain exercise. Brace continued for 3 months Full return to activities after 9–12 months when recovery of muscle strength 80–85% of cl leg	3 patients present grade 1 posterior laxity at final follow-up mean 10 m, (lesioned PCL, PLC. Lesioned ACL, PCL, PLC. Lesioned ACL, PCL, MCL, PLC). 6 p with terminal flexion restriction of 15° or more All patients satisfied with outcome at final follow-up	At 13 months mean follow-up: Lysholm score 77,4 (range 69–86), mean modified Cincinnati score 62 (range 52–72)	Mean 13 m
Sreesobh [13]	Patient 2: no recovery of CPE motor function, satisfactory sensory function	Nr	Nr	Nr	Patient 1: 3 m (died from respiratory complication). Patient 2: lost to follow-up after 9 m
Porrino[3]	Nr	Nr	Nr	Nr	Nr
Yuen[9]	Nr	Knee brace locked at full extension, passive range of motion as tolerated, non-weightbearing for 6 weeks, weight bearing and active range of motion stars after 6 weeks	Able to return running by 6 months after surgery	Nr	Nr
Williams [16]	Nr	2 weeks of limited ROM (range of motion) 0°–90°. 6 weeks non-weight-bearing. 4 months of avoiding posterior tibial sag, external rotation, open chaig hamstring exercises. Hinged knee brace, ROM from 0° to 90° 4 times a day starting on postoperative day, after 2 weeks gradually increasing as tolerated with goal of maximum flexion and extension by 6 weeks. From 6 week periodized muscular endurance, streghtn, balance, agility	6 months: stability to anterior tibial translation, stable PTFJ, no pain or instability, varus stress radiograph 0 mm side to side difference in lateral compartment gapping. PTFJ stable to anteroposterior translation on physical examination. 1 + Lachman with firm endpoint, KT-1000® 1mm of increased tibial translation compared to cl	Nr	6 m reported
Zeiton [18]	Nr	Non weight bearing on crutches for 12 weeks. Graded rehabilitation program initiating at 2 weeks with a hinged knee brace, increasing flexion gradually by 15° per week	At 6 months hill walking and jogging	Nr	Nr

*ACL* anterior cruciate ligament, *Cl* contralateral, *CPE* common peroneal nerve, *IKDC* International Knee Documentation Committee, *LCL* lateral collateral ligament, *M* months, *MCL* medial collateral ligament, *Nr* non reported, *P* patient, *PCL* posterior cruciate ligament, *PLC* posterolateral corner, *PTFJ* proximal tibiofibular joint, *Rec* reconstruction, *ROM* range of movement.

Overall, the included studies demonstrated that while PLC reconstruction techniques such as Arciero and LaPrade [11, 17] are effective in isolation, untreated PTFJ instability can compromise surgical success. Despite promising technical adaptations and favorable short-term outcomes, the current body of literature is limited by methodological heterogeneity, small sample sizes, and a lack of standardized outcome reporting.

## Discussion

The management of isolated posterolateral corner and proximal tibiofibular joint injuries is relatively well-defined in current literature, particularly for high-grade PLC injuries, where anatomical reconstruction techniques are consistently supported. However, the optimal surgical strategy for combined PLC and PTFJ injuries remains unclear. Anatomical PLC reconstructions require graft passage through tunnels in the fibular head; however when the fibular head is unstable due to a concomitant PTFJ injury, the integrity of the PLC reconstruction may be compromised. This systematic review highlights the critical importance of addressing PTFJ instability in complex knee injuries requiring PLC reconstruction.

Notably, biomechanical data from Treme et al. [11] demonstrated that although the LaPrade and Arciero techniques effectively restore stability in isolated PLC injuries, they are insufficient in the presence of PTFJ instability, reinforcing the notion that PTFJ stability is integral to successful PLC reconstruction.

A recurrent theme across the literature is the frequent underdiagnosis of PTFJ injuries, especially in the context of multiligament knee injuries. This is largely due to subtle clinical presentations and the common prioritization of more apparent injuries. Most authors rely on clinical examination, often performed under anesthesia, to identify PTFJ instability [7, 9, 14, 15]. However, the lack of specific clinical signs means these injuries often go unnoticed. Typical indicators, such as lateral knee pain with fibular head palpation, a bony prominence laterally, or anteroposterior laxity relative to the contralateral limb, should raise suspicion of a PTFJ injury [4, 8]. Early recognition is essential to avoid missed diagnoses and optimize surgical outcomes [11–13].

Magnetic resonance imaging (MRI) can be valuable in confirming PTFJ instability. Burke et al. emphasized MRI’s utility in visualizing the tibiofibular ligaments and detecting tears in acute and chronic instability [14]. Their work supports the need for dedicated imaging review in patients with acute knee trauma, particularly when PLC injury is suspected.

Mechanistically, motor vehicle accidents are the most frequently reported cause of simultaneous PLC and PTFJ injury [7, 9, 13–15]. These injuries are often accompanied by ipsilateral knee ligamentous disruptions and fractures of the femur, tibia, or patella [2, 7, 13–16, 18], further complicating management.

Surgical management strategies across the included studies varied widely. Most authors employed an open lateral approach, often assisted arthroscopically, as described by Jabara [7], Yuen [9], Williams [16], and Zeiton [18]. Techniques for PTFJ stabilization ranged from suture constructs [7], screw fixation (most commonly used) [2, 7, 14, 15], and K-wires [15], to direct ligamentous reconstruction [16]. While screw fixation appears most prevalent, the limited number of studies and lack of standardization make it impossible to define a preferred method. Instead, the choice of stabilization technique appears largely surgeon-dependent.

Outcome reporting was highly heterogeneous. Only two studies included validated patient-reported outcome measures [7, 15], while others described success in terms of physical examination findings, range of motion, pain, return to sport, or imaging results [7, 9, 11, 15, 16, 18]. This lack of consistency limits comparisons between techniques. Nevertheless, outcomes were generally positive when both PLC reconstruction and PTFJ stabilization were performed, suggesting that the act of stabilizing the PTFJ, rather than the specific technique, may be the key determinant of success.

Reported complications included common peroneal nerve palsy, hardware-related issues requiring screw removal, infections, arthrofibrosis, and PLC reconstruction failure [7, 13]. While infrequent, these underscore the technical demands and importance of meticulous surgical planning in managing these injuries.

Overall, the literature, though limited and of low methodological quality, supports the conclusion that PTFJ stability is critical for successful PLC reconstruction in combined injuries. However, the variability in diagnosis, surgical approach, and reporting standards precludes the establishment of evidence-based treatment guidelines. Future research should prioritize higher-level studies with standardized evaluation criteria to clarify best practices.

Importantly, the review underscores the potential value of integrating PTFJ stabilization into PLC reconstruction techniques. Given that the grafts used in anatomical PLC reconstruction traverse the fibular head, surgical designs that replicate the pathways of the anterosuperior and posterosuperior tibiofibular ligaments could address both injuries simultaneously. This would eliminate the need for separate fixation procedures, potentially preserving physiological PTFJ motion, reducing implant burden, and minimizing tunnel conflict. Such an approach could also prove especially advantageous in chronic cases, where healing potential of the native PTFJ ligaments is diminished.

Incorporating tibiofibular ligament reconstruction into PLC procedures may improve construct stability, streamline surgery, and reduce complications. Further biomechanical and clinical studies are warranted to evaluate such techniques and determine whether they offer superior outcomes in managing this challenging injury combination.

This review is limited by the overall scarcity and low methodological quality of the available literature on combined posterolateral corner and proximal tibiofibular joint injuries. Most included studies were case reports or small retrospective series, lacking control groups, standardized outcome measures, and consistent follow-up durations. The heterogeneity in surgical techniques, injury patterns, and reporting standards precluded quantitative synthesis and direct comparison between interventions. Additionally, the limited use of validated functional outcome scores and the predominance of descriptive or anecdotal results further restrict the generalizability of findings. These limitations highlight the need for higher-level, prospective research with standardized diagnostic criteria and treatment protocols.

On the road ahead, further development and testing of reconstructive techniques are needed to achieve optimal stabilization of both posterolateral corner and the proximal tibiofibular joint simultaneously. Such approaches should aim to achieve a stable and reliable construct, facilitating the return to normal patient knee function without sequelae, while minimizing procedure-related complications, including the number of bone tunnels, graft length required, nerve lesion, among others. In the pursuit of developing reconstruction techniques of this nature, 3D printing emerges as a valuable tool. This technology enables the creation of models representing various reconstruction approaches and facilitates stability testing under the application of forces to the joint. Compared to cadaver studies, 3D printing is more cost-effective and accessible, while offering greater anatomical accuracy and realism than studies conducted on animals. Moreover, it provides high reproducibility, as identical 3D-printed models can be produced in multiple iterations. This allows for objective comparisons aimed at determining a stable construct, without subjecting patients to surgical procedures for which reliable outcome data are not yet available.

## Conclusion

The findings of this systematic review underscore the critical importance of recognizing and stabilizing proximal tibiofibular joint instability in the context of posterolateral corner reconstruction. Failure to address PTFJ instability may compromise the integrity of anatomical PLC reconstruction techniques and negatively impact knee stability. Despite the diversity of surgical approaches reported, outcomes were generally favorable when PTFJ stabilization accompanied PLC reconstruction, suggesting that achieving joint stability, regardless of specific technique, is essential. Given the frequent underdiagnosis of PTFJ injuries, especially in multiligament knee injuries, clinicians should maintain a high index of suspicion and conduct thorough intraoperative assessments. Future research should aim to define optimal treatment strategies through methodologically rigorous studies and explore integrated reconstructive techniques that address both PLC and PTFJ instability in a single construct.

## Data Availability

No datasets were generated or analysed during the current study.
